# Reduced-Dose Regorafenib for Recurrent Glioblastoma: A Safety and Outcome Analysis

**DOI:** 10.3390/cancers18152522

**Published:** 2026-08-06

**Authors:** Massimiliano Domenico Rizzaro, Claudia Fanizzi, Giorgio Fiore, Luigi Gianmaria Remore, Guido Del Vecchio, Elena Scagliotti, Giovanni Pratelli, Stefano Borsa, Stefania Elena Navone, Ilaria Bertorelli, Luca Enrico Sironi, Gabriella Roda, Giovanni Marfia, Manuela Caroli, Marco Locatelli

**Affiliations:** 1 Department of Pathophysiology and Transplantation, University of Milan, 20122 Milan, Italy; 2 Neurosurgery Unit, Fondazione IRCCS Ca’ Granda Ospedale Maggiore Policlinico, 20122 Milan, Italy; manuela.caroli@policlinico.mi.it (M.C.); 3 Neuroradiology Unit, Fondazione IRCCS Ca’ Granda Ospedale Maggiore Policlinico, 20122 Milan, Italy; 4 Center for Aerospace Medicine and Advanced Therapies—CeMATA—Laboratory of Experimental Neurosurgery and Cell Therapy, Unit of Neurosurgery, Fondazione IRCCS Ca’ Granda Ospedale Maggiore Policlinico, 20122 Milan, Italy; 5 Department of Pharmaceutical Sciences, University of Milan, 20122 Milan, Italy; 6 Department of Biomedical, Surgical and Dental Sciences, University of Milan, 20122 Milan, Italy

**Keywords:** recurrent glioblastoma, regorafenib, reduced dose, treatment tolerability

## Abstract

Background/Objectives: Recurrent glioblastoma has a poor prognosis and no universally accepted standard of care. Regorafenib has been investigated in this setting, but its use may be limited by treatment-related toxicity. This study evaluated the feasibility, safety, and clinical outcomes of reduced-dose regorafenib in patients with recurrent IDH-wildtype glioblastoma. Methods: We retrospectively analyzed 21 patients with recurrent IDH-wildtype glioblastoma (WHO 2021) treated at a single center after progression according to the Stupp protocol. Regorafenib was started at 80 mg/day on the standard 3-weeks-on/1-week-off schedule. We assessed overall survival from diagnosis (OS1) and from first progression (OS2), progression-free survival from diagnosis (PFS1) and from first progression (PFS2), treatment exposure, dose modifications, and treatment-related adverse events (CTCAE v5.0). Results: Median PFS2 was 5 months (95% CI, 3–8), median OS2 was 6 months (95% CI, 5–10), and median OS1 was 21 months (95% CI, 15–27). No grade ≥ 3 adverse events occurred, and no patient discontinued treatment permanently because of toxicity. Conclusions: In this retrospective single-center cohort, reduced-dose regorafenib was feasible and well tolerated in selected patients. These descriptive, hypothesis-generating findings warrant prospective studies to define the role of individualized regorafenib dosing in recurrent glioblastoma.

## Simple Summary

Glioblastoma is an aggressive brain tumor that almost always returns after standard treatment. Once it recurs, the options are few, and an important goal is to control the disease while preserving neurological function and avoiding severe side effects. Regorafenib is a promising treatment for recurrent glioblastoma, but its side effects can limit its use at the standard dose. In this single-center retrospective study, we gave a reduced dose of regorafenib to selected patients with recurrent IDH-wildtype glioblastoma. The treatment was well tolerated, and no severe treatment-related side effects occurred. These findings cannot show that the reduced dose works as well as the standard dose, but they suggest that tailoring the dose to each patient may improve tolerability. Larger prospective studies are needed to confirm this.

## 1. Introduction

Glioblastoma (GBM) is the most common primary malignant brain tumor [1], with an estimated incidence of approximately 3 cases per 100,000 individuals per year in most countries of North America and Europe [2]. It carries one of the poorest prognoses of any malignancy, with a median survival of approximately 15 months [3]. Despite recent progress in targeted therapies and immunotherapy [4,5], the Stupp protocol remains the standard associated with the most favorable survival outcomes. This regimen combines a 6-week course of concomitant radiotherapy and temozolomide (TMZ) after gross total or subtotal resection with 6 cycles of high-dose adjuvant TMZ [6]; extension beyond 6 cycles is common in selected patients. No approach has yet proven superior to the Stupp protocol, and GBM eventually recurs in approximately 90% of patients [7]. Only 20–30% of those with recurrent disease are candidates for repeat resection [8], and overall survival (OS) after recurrence rarely exceeds 9 months [9].

In 2010, the Response Assessment in Neuro-Oncology (RANO) Working Group introduced standardized response criteria for high-grade gliomas (RANO-HGG), updated in 2023 [10]. Alongside definitions of complete and partial response and stable disease, these criteria delineate disease progression using combined radiological and clinical parameters. Only a few prognostic factors influence survival in recurrent GBM; among them, age, Karnofsky Performance Status (KPS), molecular profile, and extent of initial resection carry both prognostic and predictive value [11]. No first-line therapy has emerged as a universally accepted standard, few randomized trials have addressed the optimal strategy, and most evidence derives from retrospective or non-randomized studies. Tumor recurrence management must therefore be individualized, balancing risks, benefits, and quality of life in this non-curative setting [8].

Lomustine has historically been one of the most frequently used and evaluated options for recurrent glioblastoma, both as monotherapy and as a comparator in clinical trials [12], but it causes substantial myelotoxicity and achieves limited progression-free survival [12,13]. In 2018, the REGOMA trial, a multicenter, open-label, randomized, controlled phase II study, compared regorafenib with lomustine in 119 patients with recurrent GBM (60 lomustine, 59 regorafenib) [14]. Regorafenib showed a statistically significant survival advantage, with a median OS of 7.4 versus 5.6 months, although median PFS was similar (2.0 versus 1.9 months) [14]. More recently, however, the phase II/III GBM AGILE platform trial did not confirm a survival advantage for regorafenib over control treatment [15].

Regorafenib is an oral multikinase inhibitor with antiangiogenic, stromal, and oncogenic targets, providing a biological rationale for its use in glioblastoma [16,17,18,19]. Although a targeted rather than a conventional chemotherapeutic agent, it carries clinically relevant toxicity, particularly hepatotoxicity, and is often poorly tolerated: in REGOMA, 56% of regorafenib-treated patients experienced grade ≥ 3 adverse events according to the Common Terminology Criteria for Adverse Events (CTCAE) [14,20], frequently requiring hospitalization and impairing quality of life. In 2024, the REGOMA-OSS study, a large multicenter real-world analysis, confirmed the REGOMA results in 192 patients with recurrent GBM, reporting grade ≥ 3 adverse events in 22.6% of cases [21].

In the present study, we retrospectively evaluated the feasibility, safety, treatment exposure, and clinical outcomes of reduced-dose regorafenib in patients with recurrent IDH-wildtype glioblastoma treated at our institution.

## 2. Materials and Methods

### 2.1. Patient Selection

Patients were identified from the institutional neuro-oncology database over the period 2018–2025. During this period, 29 patients with recurrent IDH-wildtype glioblastoma treated with regorafenib at our institution were screened. Eight patients received standard-dose regorafenib at 160 mg/day and were excluded from the present reduced-dose analysis. The final study cohort included 21 patients who underwent surgical treatment at our institution, experienced disease progression after completion of the Stupp protocol, as defined by RANO criteria, and started regorafenib at a reduced dose of 80 mg/day.

**Figure 1:**
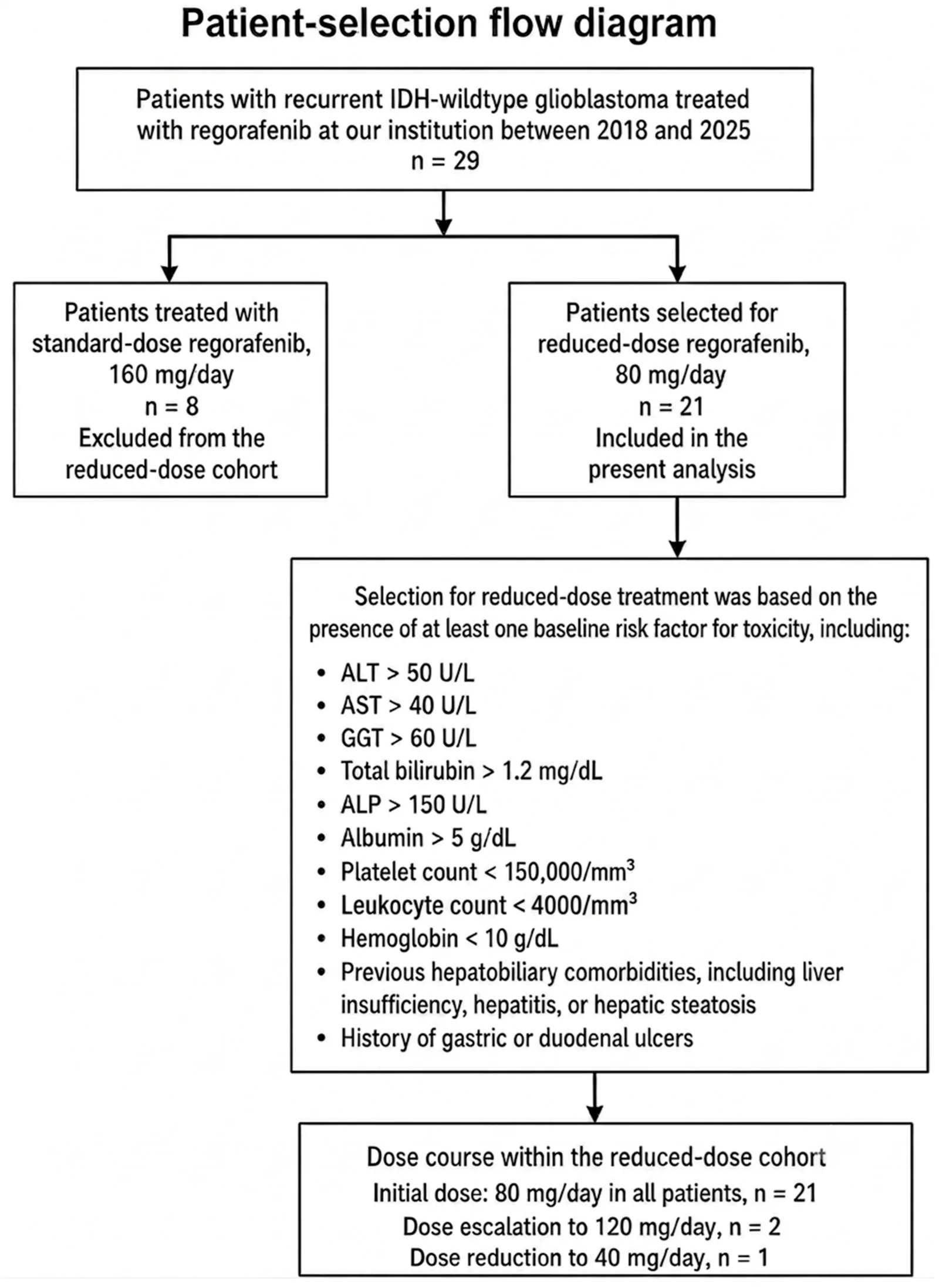
Flow diagram showing the selection of patients with recurrent IDH-wildtype glioblastoma treated with regorafenib at our institution.

Selection for the reduced-dose strategy was based on the presence of at least one baseline risk factor for toxicity, including ALT > 50 U/L, AST > 40 U/L, GGT > 60 U/L, total bilirubin > 1.2 mg/dL, ALP > 150 U/L, albumin > 5 g/dL, platelet count < 150,000/mm^3^, leukocyte count < 4000/mm^3^, hemoglobin < 10 g/dL, previous hepatobiliary comorbidities, including liver failure, hepatitis, or hepatic steatosis, or a history of gastric or duodenal ulcers. The patient-selection process is summarized in Figure 1.

Patients who were not eligible for radical tumor resection and whose diagnosis was obtained exclusively by biopsy were excluded. This criterion was applied to obtain a more homogeneous surgical cohort and to limit confounding from the unresectable disease burden and poorer prognosis typical of biopsy-only management.

All included cases had a histopathological diagnosis of glioblastoma or CNS WHO grade 4, and were IDH-wildtype according to the WHO 2021 classification. IDH status was assessed by IDH1 R132H immunohistochemistry and, when required, by molecular analysis of IDH1/2. From a molecular standpoint, the absence of IDH1/2 gene mutations was considered the sole mandatory criterion for inclusion. MGMT promoter methylation was assessed by pyrosequencing and tumors were considered methylated when the methylation level was >9%, according to the institutional molecular pathology cut-off and the previous pyrosequencing-based literature. All patients with recurrent glioblastoma were included regardless of MGMT promoter methylation status. No stratification or comparative analyses were performed for genetic alterations other than IDH1/2.

### 2.2. Outcomes

Clinical outcomes included overall survival from diagnosis (OS1) and from first progression (OS2), as well as progression-free survival from diagnosis (PFS1) and from first progression (PFS2). Treatment exposure was assessed by recording the number of completed cycles, dose modifications, treatment interruptions, and reasons for discontinuation. The severity of regorafenib-related adverse events was assessed using CTCAE version 5.0. Clinical status was longitudinally monitored using the Karnofsky Performance Status (KPS) scale. Because regorafenib was generally initiated shortly after radiological confirmation of progression, an exploratory sensitivity analysis was also performed using regorafenib initiation as time zero.

### 2.3. Postoperative Management

Approximately one month after surgery, patients received radiotherapy (60 Gy delivered in 30 fractions of 2 Gy) with concomitant low-dose temozolomide (75 mg/m^2^) for 6 weeks. This was followed by adjuvant high-dose temozolomide (150 mg/m^2^) administered for 5 consecutive days every month. Follow-up brain MRI was performed every 3 months. Progression was assessed by contrast-enhanced brain MRI according to RANO criteria and confirmed through multidisciplinary neuro-oncology review integrating radiological findings, corticosteroid use, neurological status, and clinical course. When radiological progression was suspected within the early post-radiotherapy period or when pseudoprogression was considered possible, progression was confirmed by FET-PET and advanced MRI sequences, including perfusion, diffusion and spectroscopy, before initiation of regorafenib.

At confirmed progression, patients were started on regorafenib following the REGOMA schedule (3 weeks on, 1 week off), with an initial dose of 80 mg/day. Blood tests, including liver and renal function panels and complete blood counts, were performed every two weeks. Dose escalation to 120 mg/day was considered in selected patients showing good clinical tolerance, clinical stability, absence of treatment-related adverse events, and stable liver function parameters during the first treatment cycles. Dose reduction was performed in case of persistent laboratory abnormalities or clinically relevant toxicity. Patients who underwent repeat surgery and/or re-irradiation at recurrence were also included in the analysis.

Corticosteroid use at regorafenib initiation was recorded, including a daily dexamethasone-equivalent dose when available. During treatment, steroid dose was adjusted according to neurological symptoms, edema-related radiological findings, and clinical judgment.

### 2.4. Statistical Analyses

This study was designed as a retrospective analysis of an institutional database in which clinical data, particularly treatment tolerance and adverse events, were prospectively recorded during patient follow-up. Continuous variables were summarized using median values and 95% confidence intervals or interquartile ranges, as appropriate. Categorical variables were reported as frequencies and percentages. Survival outcomes were estimated using the Kaplan–Meier method, and median OS and PFS were reported with 95% confidence intervals. Exploratory sensitivity analyses were performed according to associated treatments, including re-resection, re-irradiation, and extended temozolomide exposure. Given the small sample size, these analyses were descriptive and not powered for formal subgroup comparisons.

No formal statistical comparison with REGOMA, REGOMA-OSS, or other external cohorts was performed. This choice was made because of the retrospective single-center design, differences in sample size and patient selection, baseline characteristics, surgical management, and treatment setting between our cohort and previously published multicenter studies. Published regorafenib studies were therefore used only as external clinical context in the Discussion. All statistical analyses and Kaplan–Meier survival curve generation were performed using SPSS software (version 27.0; IBM Corp., Armonk, NY, USA).

### 2.5. Ethics Statement

The study was conducted in accordance with the Declaration of Helsinki and was approved by the local Ethics Committee (IRB#1670/2015). Given the retrospective nature of the study, the requirement for written informed consent was waived.

## 3. Results

### 3.1. Demographic and Clinical Characteristics

The study cohort consisted of 21 patients, including 5 women and 16 men (24% female, 76% male). The median age at diagnosis was 59 years. All patients had a preoperative Karnofsky Performance Status (KPS) greater than 70. This was not an inclusion criterion, but rather a baseline characteristic observed in the final study cohort. All analyzed glioblastomas were located in the supratentorial compartment.

Two of the 21 patients (9.5%) presented with multifocal disease at diagnosis; in these cases, surgical treatment included resection of all tumor foci accessible through the same surgical approach. Tumor localization was frontal in 48% of cases, temporal in 29%, and parietal in the remaining 23%.

All patients were receiving corticosteroid therapy at the time of disease progression and before regorafenib initiation. The median dexamethasone-equivalent dose at regorafenib initiation was 16 mg/day. During treatment with regorafenib, corticosteroids were gradually tapered in all patients when clinically feasible, according to neurological status and radiological findings. Corticosteroid therapy was subsequently reintroduced or increased at further disease progression, as all patients eventually experienced progression and/or death during follow-up.

According to the WHO 2021 classification, none of the tumors harbored IDH1 or IDH2 mutations. Methylation of the MGMT promoter greater than 9% was observed in 15 of 21 patients (71%). Among methylated tumors, the mean MGMT methylation level was 27%. Throughout the postoperative course, during the Stupp protocol, and at the time of progression, no patient experienced a decline in KPS below 70 (Table 1).

### 3.2. Survival

Seventy-one percent of patients completed the 6 cycles of temozolomide planned in the Stupp protocol. Among these, 47% completed 12 cycles, and in two patients continuation beyond 12 cycles was deemed clinically appropriate. Overall, patients completed a mean of 8 cycles of temozolomide before disease progression.

The median progression-free survival from diagnosis (PFS1) was 11 months (95% CI, 9–13). Four of 21 patients (19%) underwent repeat gross total tumor resection at recurrence, while 48% received re-irradiation; two of the four reoperated patients also underwent adjuvant radiotherapy. In patients undergoing re-resection, regorafenib was started approximately two weeks after surgery. In patients receiving re-irradiation, regorafenib was administered concomitantly with radiotherapy. Patients undergoing re-resection, re-irradiation, or extended temozolomide exposure were included in the analysis in order not to further reduce the sample size.

**Figure 2:**
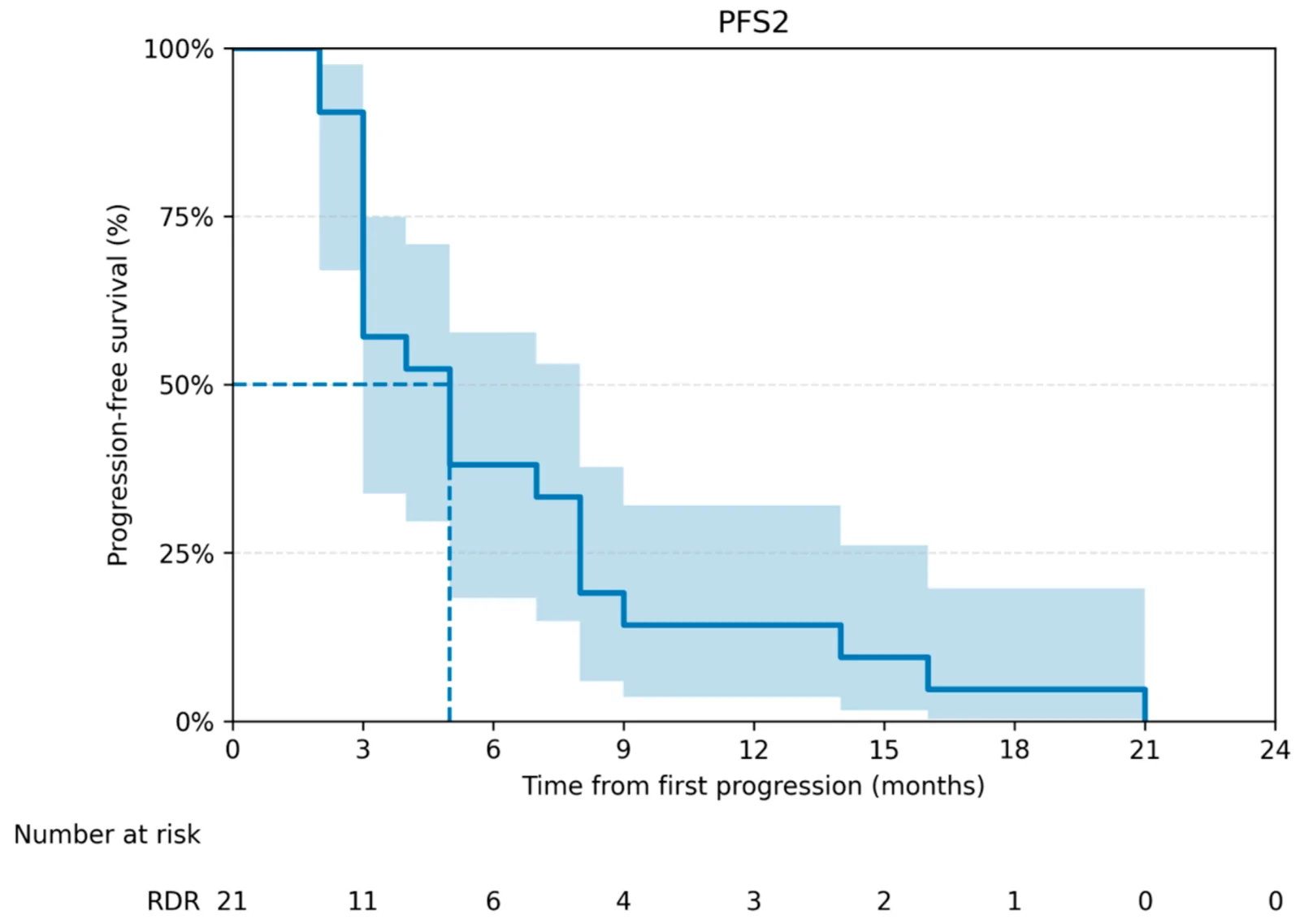
Kaplan–Meier curves depicting progression-free survival (PFS2) of our patients. Median times are indicated by dashed lines. Shaded regions represent the 95% confidence interval. Abbreviations: RDR, reduced-dose regorafenib.

**Figure 3:**
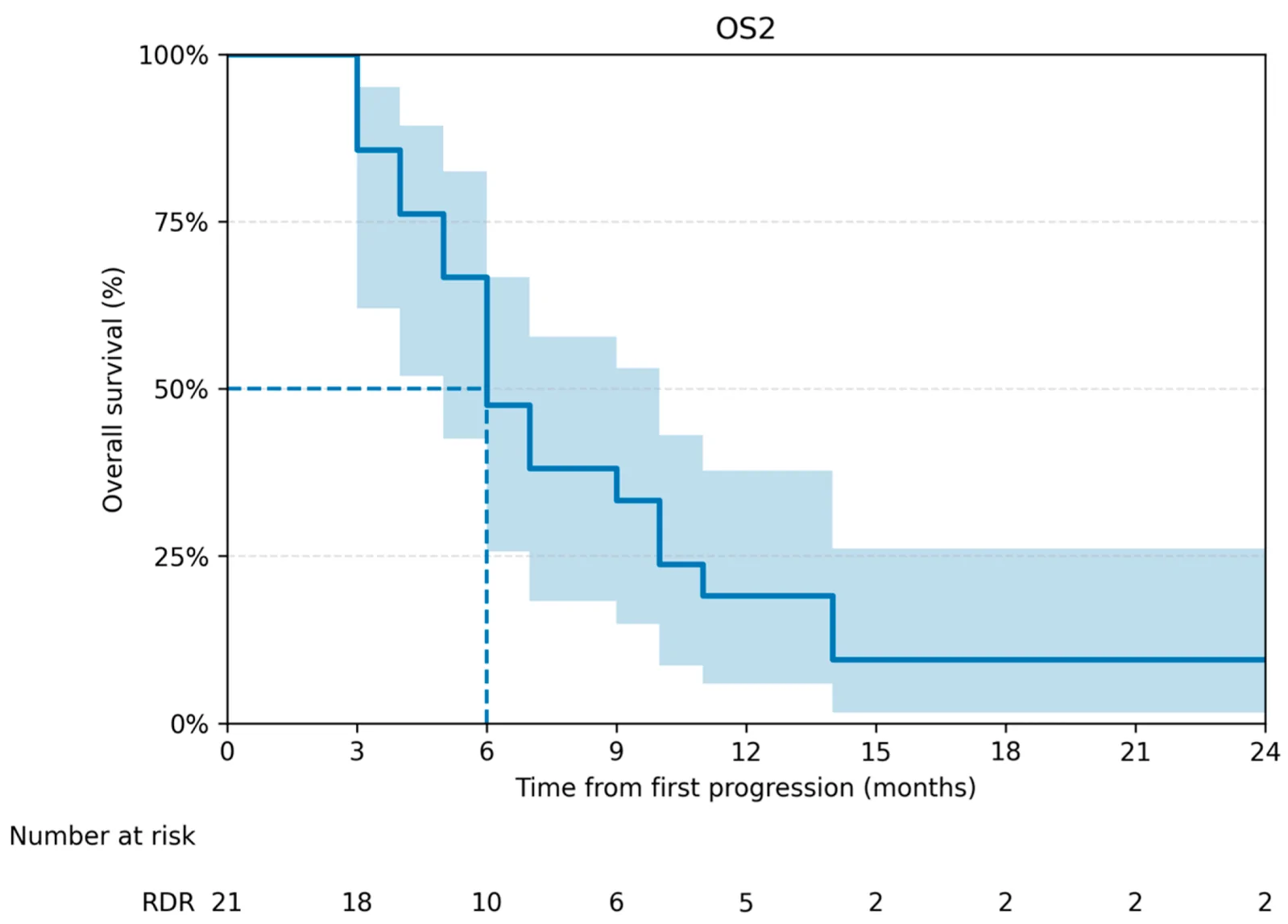
Kaplan–Meier curves depicting overall survival (OS2) of our patients. Median times are indicated by dashed lines. Shaded regions represent the 95% confidence interval. Abbreviations: RDR, reduced-dose regorafenib.

Median progression-free survival after first progression (PFS2) was 5 months (95% CI, 3–8), with a median overall survival after first progression (OS2) of 6 months (95% CI, 5–10). Median overall survival from diagnosis (OS1) was 21 months (95% CI, 15–27). Kaplan–Meier curves for OS2 and PFS2 in the study cohort are shown in Figure 2 and Figure 3. A summary of the results obtained in our patient cohort is shown in Table 2.

Regorafenib was started approximately two weeks after the MRI documenting disease progression in all patients. To assess whether this interval influenced survival estimates, an exploratory sensitivity analysis was performed using regorafenib initiation as time zero instead of radiological progression. In this analysis, median PFS2 and median OS2 showed no substantial change compared with the estimates calculated from first progression. An exploratory sensitivity analysis according to associated treatments is reported in Table 3. Median PFS2 remained broadly within the same range across subgroups. Median OS2 was numerically longer in patients who underwent re-resection or re-irradiation, or received more than 6 cycles of adjuvant temozolomide; however, confidence intervals were wide, and these analyses were underpowered because of the small number of patients in each subgroup. Therefore, these findings were considered descriptive and did not change the overall interpretation of the study.

All patients included in the study died during the observation period. After second progression, two patients maintained a KPS greater than 50, allowing continuation of treatment: one patient received an additional course of hypofractionated radiotherapy, while the other was treated with lomustine.

### 3.3. Treatment Exposure

All patients started regorafenib after radiologically confirmed first progression. The planned schedule was 3 weeks on treatment followed by 1 week off treatment, with a starting dose of 80 mg/day. The median number of completed cycles was 4 (IQR 3–7.5).

Dose escalation to 120 mg/day was performed in two patients. In one patient, the dose was increased after two months of treatment, while in the other patient after one month. In both cases, escalation was based on good clinical tolerance, clinical stability, absence of treatment-related adverse events, and stable liver function parameters. Both patients remained on the increased 120 mg/day dose until disease progression.

Dose reduction to 40 mg/day was required in one patient after the second cycle because of persistent CTCAE grade 2 transaminase elevation. The alteration persisted for one cycle and improved after dose reduction. No patient required permanent treatment discontinuation because of liver toxicity. Discontinuation was instead driven by disease progression. A detailed summary of treatment exposure is reported in Table 4. Median treatment duration was 4 months (IQR, 3–7), and median cumulative dose was 6720 mg (IQR, 5040–11,760). Median relative dose intensity was 100% (range, 83.3–143.8). No treatment interruptions occurred, and adherence was 100% according to institutional treatment records.

### 3.4. Adverse Events

Grade 1 and grade 2 adverse events according to the CTCAE scale were observed in 9% and 29% of patients, respectively. The most frequently reported symptoms were fatigue (37%), abdominal pain (12%), petechiae (12%), and paresthesias (12%).

Nineteen percent of patients experienced elevations in liver function parameters, including alanine aminotransferase, aspartate aminotransferase, alkaline phosphatase, gamma-glutamyltransferase, and bilirubin levels. Among these patients, 50% remained asymptomatic.

No patient in our cohort developed grade ≥ 3 adverse events, and no toxicity-related hospitalization was required. No patient required permanent treatment discontinuation due to treatment-related toxicity. A detailed summary of adverse events, including CTCAE grade, frequency, management, and outcome, is reported in Table 5.

## 4. Discussion

The main finding of this single-center study was the favorable tolerability of reduced-dose regorafenib, started at 80 mg/day, in patients with recurrent IDH-wildtype glioblastoma: no grade ≥ 3 adverse events occurred, and no patient discontinued treatment permanently because of toxicity.

Regorafenib has an established role in several solid tumors, including metastatic colorectal cancer [22], gastrointestinal stromal tumors [23], and hepatocellular carcinoma [24], but its role in recurrent glioblastoma remains controversial. In Italy, it has been reimbursed for recurrent glioblastoma since 2020, following the REGOMA trial.

The survival benefit shown for regorafenib over lomustine in REGOMA [14] was supported by real-world data such as REGOMA-OSS [21], but was not confirmed by the phase II/III GBM AGILE platform trial [15]. Its role in recurrent glioblastoma therefore remains debated, and further studies are needed to identify the patients most likely to benefit.

In our cohort, median PFS2 and OS2 were 5 and 6 months, respectively. These results should be interpreted within the generally poor prognosis of recurrent glioblastoma, where survival after recurrence is usually limited and rarely exceeds 9 months. Our findings therefore provide descriptive clinical context rather than evidence of comparative efficacy. No formal comparison with REGOMA or REGOMA-OSS was performed given the methodological differences between cohorts.

Tolerability is central to the management of recurrent glioblastoma. Regorafenib causes clinically relevant adverse events, including hand–foot skin reaction, rash or desquamation, stomatitis, diarrhea, hypertension, liver dysfunction, and fatigue [25,26]. The high rates of grade ≥ 3 events reported in REGOMA and REGOMA-OSS [14,21] underline the importance of dosing strategies that reduce toxicity, particularly in a non-curative setting. In our cohort, adverse events were mild to moderate and were managed in the outpatient setting or through routine laboratory monitoring. Dose reduction was required in only one case because of persistent elevation of liver function parameters. No patient required hospitalization for treatment-related toxicity. The absence of severe toxicity and of toxicity-related discontinuation may be clinically meaningful in recurrent glioblastoma, where preserving neurological function and minimizing treatment burden are key goals.

Many commonly used drugs alter hepatic cytochrome P450 3A4 metabolism and can reduce the efficacy or increase the toxicity of tyrosine kinase inhibitors [27,28]; this is particularly relevant in glioblastoma, where patients frequently receive antiepileptic drugs, corticosteroids, anticoagulants, and other supportive therapies. In our cohort, no patient was taking medications with clinically relevant regorafenib interactions.

Alternative dosing strategies have been explored to improve regorafenib tolerability. In neuro-oncology, escalation from 80 mg/day to 120 mg/day and 160 mg/day in the absence of significant toxicity has been reported, including by Rudà et al. [29], on the rationale that early tolerability identifies patients able to sustain higher cumulative exposure while sparing more vulnerable ones. In metastatic colorectal cancer, the RETRITA study found that escalation from 80 mg/day only in patients with good tolerability prolonged progression-free survival and reduced severe adverse events compared with fixed dosing, with comparable overall survival [30]. Although derived from a different tumor type, these findings support the concept that individualized regorafenib dosing may improve tolerability without compromising clinical activity. Our approach differed from formal escalation, as most patients were maintained at the reduced starting dose according to baseline clinical and hepatic risk.

Several limitations should be acknowledged. First, this was a retrospective, single-center analysis including only 21 patients. Second, no contemporaneous internal cohort of standard-dose regorafenib patients was available, so the study was designed as descriptive, with REGOMA and REGOMA-OSS serving only as external clinical context. Third, our cohort was highly selective, as all patients had preserved KPS, although this was not a formal inclusion criterion; all had undergone surgical resection at diagnosis; biopsy-only patients were excluded; MGMT promoter methylation was frequent; and a substantial proportion received re-resection and/or re-irradiation at recurrence. In addition, the relatively low median age of the cohort and the use of corticosteroids at regorafenib initiation may have further influenced outcome interpretation. These factors may have affected survival independently of regorafenib and limit the generalizability of our findings to less selected recurrent glioblastoma populations. For this reason, our findings may be more applicable to patients with recurrent IDH-wildtype glioblastoma who share similar baseline characteristics, including preserved functional status, prior surgical resection, and clinical suitability for reduced-dose regorafenib, rather than to an unselected recurrent glioblastoma population. Although exploratory sensitivity analyses according to associated treatments were performed, these analyses were underpowered because of the small sample size and should therefore be interpreted with caution. Fourth, adverse events were retrospectively collected from clinical records, outpatient visits, treatment reports, and laboratory tests performed at our institution. Therefore, mild or patient-reported toxicities may have been underestimated. The absence of grade ≥ 3 adverse events should not be interpreted as evidence of superior safety compared with standard-dose regorafenib. Finally, no formal quality-of-life or patient-reported outcome measures were available.

Overall, our findings suggest that reduced-dose regorafenib may represent a feasible strategy in patients with recurrent IDH-wildtype glioblastoma who are considered at increased risk of toxicity. These results should be regarded as hypothesis-generating. Future prospective studies should clarify whether individualized dosing can improve tolerability while maintaining clinically meaningful activity. In patients undergoing re-resection, exploratory translational analyses could also help assess tissue drug exposure and on-target effects at reduced doses. Such data may be useful for designing more rational and tolerable combination strategies.

## 5. Conclusions

In this single-center retrospective real-world experience, reduced-dose regorafenib was feasible and showed a favorable tolerability profile in selected patients with recurrent IDH-wildtype glioblastoma. No grade ≥3 adverse events were observed, and no patient discontinued treatment because of toxicity.

These survival outcomes should be interpreted descriptively and cannot establish equivalence with standard-dose regorafenib or with previously published cohorts. Prospective studies are needed to better define the role of individualized regorafenib dosing in recurrent glioblastoma.

## Figures and Tables

**Table 1:** Baseline demographic, clinical, and molecular characteristics of the study cohort. Abbreviations: KPS, Karnofsky Performance Status; SD, standard deviation; WHO, World Health Organization; IDH, isocitrate dehydrogenase; MGMT, O6-methylguanine-DNA methyltransferase.

Characteristics	Patients (*n* = 21)
Sex, *n* (%)	
Male	16 (76)
Female	5 (24)
Age at diagnosis, years	
Median (IQR)	59 (55–62)
Preoperative KPS > 70, *n* (%)	21 (100)
Tumor location, *n* (%)	
Supratentorial	21 (100)
Dominant hemisphere involvement	11 (52)
Multifocal disease at diagnosis, *n* (%)	2 (9.5)
Lobar location, *n* (%)	
Frontal	10 (48)
Temporal	6 (29)
Parietal	5 (23)
Corticosteroid therapy at progression, *n* (%)	21 (100)
Molecular characteristics (WHO 2021)	
IDH1/IDH2 mutation, *n* (%)	0 (0)
MGMT promoter methylation > 9%, *n* (%)	15 (71)
Mean MGMT methylation level (%), mean (SD)	27 (17)

**Table 2:** Summary of the results obtained in our patient cohort. Abbreviations: OS1, overall survival from diagnosis; OS2, overall survival from first progression; PFS1, progression-free survival from diagnosis; PFS2, progression-free survival from first progression; CI, confidence interval.

Parameter	Median
OS1	21 months (95% CI, 15–27)
OS2	6 months (95% CI, 5–10)
PFS1	11 months (95% CI, 9–13)
PFS2	5 months (95% CI, 3–8)

**Table 3:** Exploratory sensitivity analysis according to associated treatments. Abbreviations: CI, confidence interval; PFS2, progression-free survival from first progression; TMZ, temozolomide.

Subgroup	*n*	Median PFS2, Months (95% CI)	Median PFS2, Months (95% CI)
Overall Cohort	21	5 (3–8)	6 (5–10)
Re-resection	4	5.5 (3–14)	8.5 (3–14)
No re-resection	17	5 (3–8)	6 (5–10)
Re-irradiation	10	5 (2.5–11)	8 (3.5–14)
No re-irradiation	11	6 (5–10)	6 (5–10)
TMZ exposure > 6 cycles	12	6.5 (3–11.5)	9.5 (4.5–12.5)
TMZ exposure < 6 cycles	9	4 (3–7)	6 (3–7)
Re-resection and/or re-irradiation	12	5 (3.5–8)	8 (4–12.5)
No re-resection and/or re-irradiation	9	5 (3–8)	6 (5–10)

**Table 4:** Treatment exposure and dose modifications during reduced-dose regorafenib. **Abbreviations:** CTCAE, Common Terminology Criteria for Adverse Events; IQR, interquartile range.

Variable	Median
Treatment duration, months	4 (IQR, 3–7)
Cumulative dose, mg	6720 (IQR, 5040–11,760)
Relative dose intensity, %	100 (range, 83.3–143.8)
Treatment interruptions	0 (0%)
Treatment adherence	100%
Dose escalation to 120 mg/day	2/21 (9.5%)
Dose reduction to 40 mg/day	1/21 (4.8%)
Reason for dose escalation	Favorable clinical and biochemical profile
Reason for dose reduction	Persistent CTCAE grade 2 transaminase elevation

**Table 5:** Treatment-related adverse events with reduced-dose regorafenib. Patients could experience more than one adverse event; therefore, percentages for individual events are not mutually exclusive. Abbreviations: CTCAE, Common Terminology Criteria for Adverse Events; GGT, Gamma-Glutamyl Transferase.

Adverse Event	CTCAE Grade	Patients, *n* (%)	Outcome	Management
Fatigue	1–2	8 (37)	No discontinuation	Observation
Abdominal pain	1–2	3 (12)	No discontinuation	Observation
Petechiae	1–2	3 (12)	No discontinuation	Observation
Limb paresthesias	1–2	3 (12)	No discontinuation	Observation
Transaminase elevation	1–2	2 (9.6)	No discontinuation	Observation, Dose reduction
Bilirubin elevation	1–2	1 (4.8)	No discontinuation	Observation
GGT elevation	1	1 (4.8)	No discontinuation	Observation

## Data Availability

The raw data supporting the conclusions of this article will be made available by the authors on request.

## Abbreviations

GBM	Glioblastoma
OS	Overall Survival
PFS	Progression-Free Survival
RANO	Response assessment in neuro-oncology
MGMT	O^6^-methylguanine-DNA methyltransferase
KPS	Karnofsky Performance Status
IDH	Isocitrate Dehydrogenase
REGOMA	Regorafenib in Relapsed Glioma
CI	Confidence Interval
TMZ	Temozolomide
RDR	Reduced-Dose Regorafenib
CTCAE	Common Terminology Criteria for Adverse Events
GGT	Gamma-Glutamyl Transferase
ALT	Alanine-Aminotransferase
AST	Aspartate-Aminotransferase
IQR	Interquartile Range
